# Multidrug resistant lymph node fistula tracheobronchial tuberculosis: A case report

**DOI:** 10.1097/MD.0000000000018288

**Published:** 2019-12-10

**Authors:** Jiang Liu, Shouning Xie, Laishun Yu, Xiaoling Su

**Affiliations:** a Graduate School of Qinghai University; b Orthopedics; c Respiratory and critical care medicine; d Cardiology Department, Qinghai Province People's Hospital, Qinghai, China.

**Keywords:** bronchoscopic cryotherapy, case report, Multidrug resistant tuberculosis, tracheobronchial tuberculosis

## Abstract

**Rationale:**

The patient in this case report has been diagnosed with multidrug resistant lymph node fistula tracheobronchial tuberculosis (TBTB). The PubMed was searched using the keywords “Tuberculosis, Multidrug-Resistant”, “Tuberculosis”, and “Bronchial Fistula”, and the results yielded no similar case reports. Therefore, this report helps us to explore more on the causes of multidrug resistance and formation of lymph node fistula, as well as associated treatment strategies.

**Patient concerns:**

A 15-year old Tibetan girl who was previously treated in the local Hospital for Infectious Diseases for repeated TBTB demonstrated poor treatment outcomes, and so was further diagnosed in our hospital. After standard treatments, the cough and expectoration of the girl showed improvement, and mycobacterium culture showed negative results. Thoracic CT showed local compression of the right bronchus, and disappearance of stenosis. Bronchoscopy showed that the fistula was closed and healed.

**Diagnoses:**

Multidrug resistant lymph node fistula TBTB.

**Interventions:**

Antituberculosis drugs such as pyrazinamide (0.75 g/time, twice per day), moxifloxacin (0.4 g per day), protionamide enteric-coated tablets (0.2 g/time, 3 times per day), pasiniazide tablets (0.3 g/time, 3 times per day), and capreomycin (0.75 g per day) were orally administered. The treatment included an 8-month intensive treatment phase and a 12-month consolidation phase. Bronchoscopic local injection combined with cryotherapy was also conducted.

**Outcomes:**

Bronchoscopy showed that the fistula was closed and healed, mycobacterium culture showed negative results, and thoracic CT showed local compression of the right bronchus, disappearance of stenosis after treatment.

**Lessons:**

(1) This girl had a history of long-term oral intake of antituberculosis drugs, but the treatment effectiveness remained poor. Therefore, resistance to tuberculosis can be considered, and also mycobacterium culture and drug sensitivity tests should be considered. After these, the treatment strategies should be adjusted accordingly.

(2) TBTB should be further classified by analyzing under the bronchoscope to decide the best treatment strategy accordingly.

## 1 Introduction

Multidrug resistant tuberculosis (MDR-TB) refers to tuberculosis (TB) caused by Mycobacterium tuberculosis infection, which has been confirmed resistant at least to both isoniazid and rifampicin in in vitro drug sensitivity test. The prevalence is 2.5%.^[1]^ Tracheobronchial tuberculosis (TBTB) refers to TB at mucosa, submucosa, smooth muscle, cartilage, or outer membrane of the trachea or bronchus. A previous study has reported that about 10% to 40% of patients with active pulmonary TB are with TBTB.^[2]^ TBTB mainly occurs in young or middle-aged women, with a male-to-female ratio of 1:2 to 1:3.^[3–5]^ TBTB is classified into 6 types by viewing under a bronchoscope, which type VI (lymph node fistula type) is a rare. However, the present case was unique for being not only MDR-TB, but also a relatively rare lymph node fistula TBTB. Patients with both these types concurrently were relatively rare, and so the case was reported here. Written informed consent was obtained. This study was a case report so ethical approval was waived or not necessary.

## 2 Case report

A 15-year-old Tibetan girl was admitted to our hospital due to aggravated intermittent cough and expectoration for 1 month. The girl was had intermittent cough and expectoration for 1 year before, without any evident causes. The girl was hospitalized in the local Hospital for Infectious Diseases in December 2017, and underwent bronchoscopy. The results revealed “secondary pulmonary TB and endobronchial TB”. However, the bronchial lavage fluid was Xpert:RIF positive, suggesting the possibility of drug resistance. The 4-drug antituberculous therapy, that is, protionamide enteric-coated tablets (0.2 g/time, 3 times per day), ethambutol (0.75 g per day), pyrazinamide (0.75 g/time, twice per day), and amikacin (intravenous dripping, 0.6 g per day) was given, which alleviated the cough and expectoration. The girl was admitted to the local Hospital for Infectious Diseases again in May 2018 due to liver dysfunction, and underwent bronchoscopy. Due to liver dysfunction and Xpert:RIF negative bronchial lavage fluid, the treatment strategy was adjusted to 3-drug antituberculous therapy, that is, levofloxacin (0.5 g per day), isoniazide (0.3 g per day), and ethambutol (0.75 g per day). Regular re-examinations in the Outpatient Department showed that the liver function returned to normal. The girl was admitted to the Outpatient Department of the West China Hospital of Sichuan University in August 2018, and the treatment was adjusted to 3-drug antibuberculous therapy, that is, rifampicin capsule (0.45 g per day), isoniazide (0.3 g per day), and ethambutol (0.75 g per day). The girl was admitted to the local Hospital for Infectious Diseases again in January 2019 due to cough, expectoration, chest distress, and breathe shortness. As the bronchial lavage fluid was Xpert:RIF positive, the treatment strategy was adjusted to 5-drug antituberculous therapy, that is, parinazid tablets (0.3 g/time, 3 times per day), protionamide enteric-coated tablets (0.2 g/time, 3 times per day), moxifloxacin (0.4 g per day), rifampicin capsule (0.45 g per day), and capreomycin (0.75 g per day). In addition, bronchoscopic injection (rifampicin injection [0.15 g per day] and isoniazide injection [0.2 g]) was also given for several times. However, the effectiveness of treatment still remained suboptimal. The girl was then admitted to our hospital for further diagnosis and treatment.

## 3 Diagnostic assessment

Physical examinations on admission revealed her blood pressure to be 120/70 mmHg. The breath sounds in bilateral lungs were coarse, with no dry or moist rales, as well as pleural friction sound was found. The heart rate of the girl was 8 beats per minute, the heart rhythm was regular, and the heart sounds were normal. No pathological cardiac souffle or pericardium scratching was heard at the auscultatory valve areas. The abdomen was flat, without any pressing pain or rebound tenderness. The liver and spleen were not touched, and the borborygmus was normal.

Laboratory and imaging examinations revealed positive for acid-fast bacillus (AFB) in the bronchial lavage fluid, mycobacterium culture showed positive results (+), and drug resistance test showed that the mycobacterium tuberculosis was resistant to rifampicin, isoniazide, and ethambutol. Blood routine examinations, biochemical examinations, procalcitonin test, erythrocyte sedimentation rate (ESR), tumor indexes, and coagulation test all showed no evidence of any abnormalities. Thoracic CT scanning showed local compression of the right bronchus, causing stenosis of the bronchial lumen. In addition, shadows of intensity of soft tissues were also observed in the bronchus.

**Figure 1:**
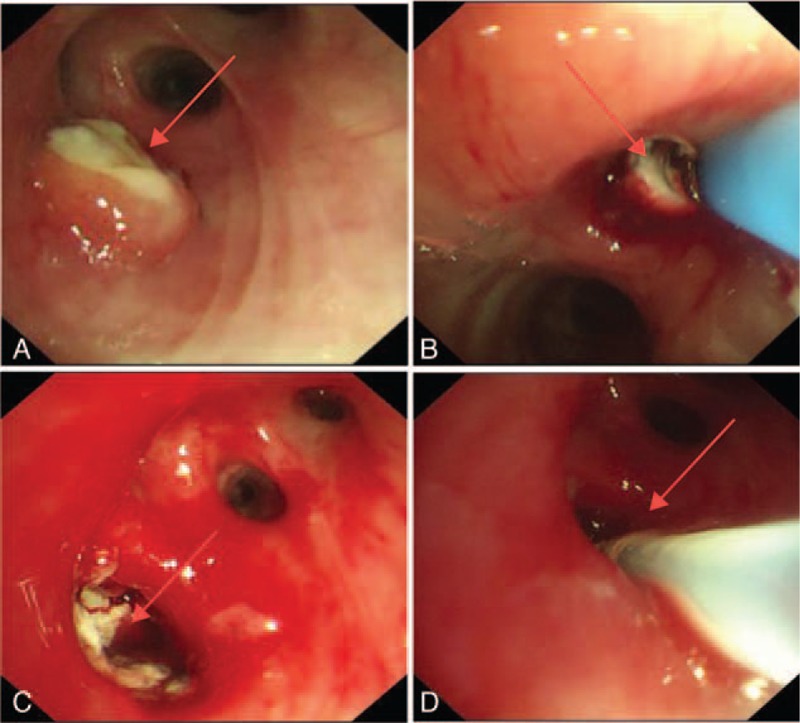
Findings of bronchoscopic examinations; A: Granuloma at the opening of the right superior lobar bronchus; B: Cryotherapy; C: The sinus tract was found; D: 0.3 g isoniazide was injected into the sinus tract.

**Figure 2:**
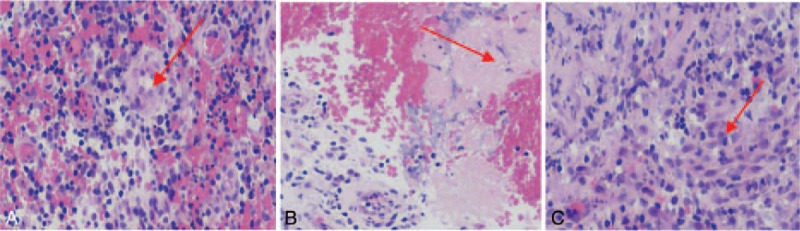
Findings of pathological examinations; A: Granulomatous lesions (×40); B: Coagulative necrosis (×40); C: Multinucleated giant cells and epithelioid cells (×40).

After admission, bronchoscopy revealed granuloma at the opening of the right superior lobar bronchus. After conducting cryotherapy, a sinus tract was observed, which was considered to be lymph node-bronchial fistula. Thus, a repeated cryotherapy as well as local injection of isoniazide (0.3 g) was performed (Fig. 1). Pathological examinations showed granulomatous lesions, coagulative necrosis, and multinucleated giant cells, suggesting TB-like changes (Fig. 2).

The final diagnosis of the lesion in the girl revealed multidrug resistant lymph node fistula TBTB.

## 4 Therapeutic intervention

Antituberculosis drugs including pyrazinamide (0.75 g/time, twice per day), moxifloxacin (0.4 g per day), protionamide enteric-coated tablets (0.2 g/time, 3 times per day), pasiniazide tablets (0.3 g/time, 3 times per day), and capreomycin (0.75 g per day) were given for treatment. The treatment included an 8-month intensive treatment phase and a 12-month consolidation phase. Liver protection treatments were also provided during the therapy using reduced glutathione tablets (0.4 g/time, 3 times per day) and bicyclol tablets (25 mg/time, 3 times per day).

Cryotherapy combined with local injection was also opted. In brief, bronchoscopy was conducted under local anesthesia. After reaching the lesion area, the cryosurgery probe was inserted through the catheter for tracheal therapy at the lesion area, and then cryoablation was performed at −69°C. Each cryoablation session lasted for 5 s, followed by an interval of 30 seconds. For one treatment session, cryoablation was conducted for 5 to 10 cycles. Next, 0.3 g of isoniazide was injected to the lesion area and then the treatment was considered complete.

## 5 Follow-up and outcomes

As the girl was living in the plateau area and her commuting was inconvenient, re-examinations every other month after discharge was suggested for her. The girl has received 4 re-examinations before her contribution to this report.

**Figure 3:**
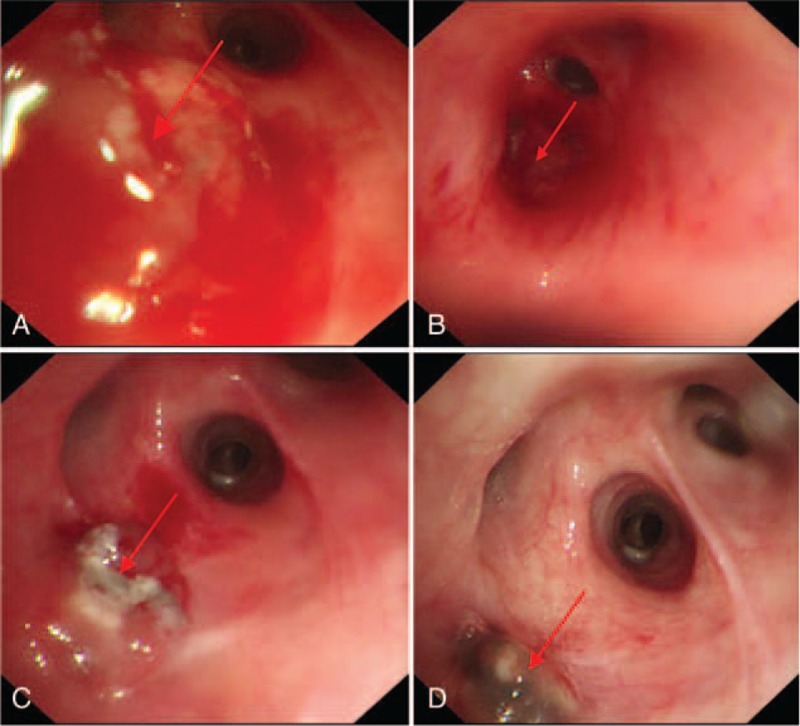
(A) Findings of bronchoscopic examinations at 1 month after discharge. (B)Findings of bronchoscopic examinations at 2 months after discharge. (C) Findings of bronchoscopic examinations at 3 months after discharge. (D)Findings of bronchoscopic examinations at 4 months after discharge.

One month after discharge: Bronchoscopic cryotherapy combined with local injection of isoniazide (0.3 g) was administered as treatment. Examinations showed mucosal hyperemia at the right superior lobar bronchus, and neogranuloma at the opening (Fig. 3A).

Two months after discharge: Bronchoscopic cryotherapy combined with local injection of isoniazide (0.3 g) was given. Examinations showed granuloma hyperplasia at the opening of the right superior lobar bronchus, which bled after gentle touch (Fig. 3B).

Three months after discharge: Bronchoscopic cryotherapy combined with local injection of isoniazide (0.3 g) was performed. Examinations showed granuloma hyperplasia at the opening of the right superior lobar bronchus, while the bronchial lumen was patent (Fig. 3C).

**Figure 4:**
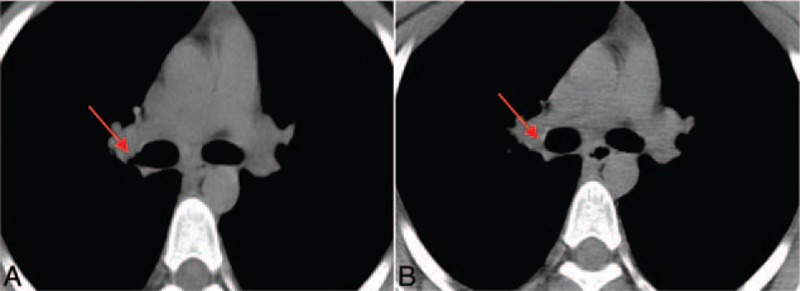
Findings of thoracic CT scanning. A: CT image before treatment. Local compression of the right bronchus caused stenosis of the bronchial lumen. In addition, shadows of intensity of soft tissues are also observed in the bronchus. B: CT image after treatment. The local compression of the right bronchus, as well as disappearance of stenosis was observed.

Four months after discharge: Bronchoscopic cryotherapy combined with local injection of isoniazide (0.3 g) was conducted. Examinations showed disappearance of inflammation at the opening of the right superior lobar bronchus, tissue repairing was observed, formation of granuloma at the orificium fistulae, and the orificium fistulae was closed and healed. Local anthracosis was absorbed (Fig. 3D). Thoracic CT showed local compression of the right bronchus, as well as disappearance of stenosis after treatment (Fig. 4). Mycobacterium culture was conducted again, which showed negative results. These findings demonstrated that the current treatment was effective.

## 6 Discussion

The girl was diagnosed with multidrug resistant lymph node fistula TBTB. PubMed was searched using the keywords “Tuberculosis, Multidrug-Resistant”, “Tuberculosis”, and “Bronchial Fistula”, but no similar cases have been reported yet. Therefore, this case was reported to further explore the causes of multidrug resistance and formation of lymph node fistula, as well as the treatment strategies. According to the *Global Tuberculosis Report* issued by WHO in 2018,^[1]^ 2.5% of the TB patients had MDR-TB/RR-TB in 2017. While among TB patients in China, 0.9% were with MDR-TB/RR-TB. These findings showed that MDR-TB was relatively rare, with only limited attention paid on it, while the treatment was not standardized. Thus, treatment duration could be very long, while the effectiveness of treatment remained poor, and the mortality rate of patients was high. In light of the previous treatments of the present case, the girl was from a high-incident area of drug resistant TB (Qinghai Plateau, Tibetan people), and had a history of long-term use of antituberculosis drugs. Both these factors were regarded as high risk factors for drug resistant TB, and were the major causes of MDR-TB in this girl. According to the treatment strategy for treating MDR by WHO in 2016,^[6]^ at least 5 effective drugs, including pyrazinamide and 4 other essential second-line anti-TB drugs should be given for treatment during the intensive treatment phase. Among the 4 essential second-line anti-TB drugs, 1 drug from group A, 1 drug from group B, and 2 drugs from group C should be chosen for treating TB, respectively. High dose isoniazide and/or ethambutol could also be added to the treatment strategy for synergistic therapeutic effects. The total treatment phase should be for 20 months, which included an 8-month intensive treatment phase and a 12-month consolidation phase. According to these recommendations, the final anti-TB treatments prescribed for the girl were as follows: pyrazinamide (0.75 g/time, twice per day), moxifloxacin (0.4 g per day), protionamide enteric-coated tablets (0.2 g/time, 3 times per day), pasiniazide tablets (0.3 g/time, 3 times per day), and capreomycin (0.75 g per day). Due to liver dysfunction after intake of pyrazinamide, the glutathione tablets (0.4 g/time, 3 times per day) and bicyclol tablets (25 mg/time, 3 times per day) were reduced in order to protect the liver.

Previous studies have reported that 10% to 40% of active TB patients were accompanied with TBTB.^[2]^ The clinical diagnosis of TBTB mainly relies on bronchoscopic examinations, and also requires bronchoscopic classification. The treatments are different for different types of lesions. Bronchoscopic examinations revealed the girl with lymph node fistula TBTB, which could easily reach the airway through the TB of mediastinal or hilar lymph nodes, thus forming lymph node-bronchial fistula.^[7,8]^ In addition, thoracic CT scanning on admission also showed local compression of the right bronchus and stenosis of the bronchial lumen, which were also the major causes for the formation of sinus tract and secondary lymph node fistula. Bronchoscopiclocal injection of anti-TB drugs combined with cryotherapy can be used for treating this disease. Local administration of anti-TB drugs in bronchus could directly deliver the drugs to the lesion area, exerting anti-TB functions. In addition, drug administration in the bronchus was conducted under the assistance of bronchoscopy, which had several advantages of easy conduction, and accurate administration of the drug. While the effects of cryotherapy were relatively mild, and thus the local reactions were also mild, and so the patients could be easily tolerated. Cryotherapy could hardly damage the airway cartilage, causing airway perforation. The rates of granulation tissue hyperplasia and fibrous scar formation were very low, and does not affect the cardiac pacemaker and metal or silicone stent.^[9,10]^ Mu et al^[9]^ have also showed that bronchoscopic local injection of anti-TB drugs combined with cryotherapy demonstrated high effectiveness, but with no severe adverse reactions in treating lymph node fistula TBTB.

According to the previous treatment strategies, as well as the diagnosis and treatments provided by us, we speculated the possibility of drug resistance. The drug-resistance test of Mycobacterium tuberculosis confirmed MDR-TB in the girl. Therefore, optimal oral anti-TB treatment strategy was given accordingly. In addition, the endobronchial tuberculosis was further classified by bronchoscopic examinations, which suggested lymph node fistula TBTB. Thus, bronchoscopic local injection of anti-TB drugs combined with cryotherapy was provided for treatment. The girl was followed up regularly, and the results showed that the fistula was closed and healed, and the Mycobacterium culture showed negative results. These findings indicated that the current treatment was effective. Regular follow-up was instructed for the girl to help us understand the changes of the disease conditions. However, there are also some drawbacks in the treatment of this girl. For instance, the interval between follow-up visits was relatively long, while local injection of anti-TB drugs combined with cryotherapy for lymph node fistula TBTB should be conducted every other week. As the girl was living in a high plateau area with inconvenient commuting, treatment could not be conducted every other week.

## 7 Conclusion

The findings showed that oral administration of pyrazinamide (0.75 g/time, twice per day), moxifloxacin (0.4 g per day), protionamide enteric-coated tablets (0.2 g/time, 3 times per day), pasiniazide tablets (0.3 g/time, 3 times per day), and capreomycin (0.75 g per day) for 20 months (including an 8-month intensive treatment phase and a 12-month consolidation phase), and in combination with bronchoscopiclocal injection of anti-TB drugs with cryotherapy were considered effective for treating MDR lymph node fistula TBTB.

## Acknowledgments

We sincerely thank the individuals that contributed to this case report. There are no conflicts of interests of the authors.

## Author contributions

**Data curation:** Jiang Liu, Shouning Xie, Xiaoling Su.

**Formal analysis:** Jiang Liu, Shouning Xie.

**Funding acquisition:** Shouning Xie.

**Methodology:** Laishun Yu.

**Project administration:** Xiaoling Su.

**Writing – original draft:** Jiang Liu, Shouning Xie.

**Writing – review & editing:** Laishun Yu.
